# Supplementary material for: miR-125-3p and miR-276b-3p Regulate the Spermatogenesis of Bactrocera dorsalis by Targeting the orb2 Gene
Source: Genes (Basel). 2022 Oct 15;13(10):1861. doi: 10.3390/genes13101861 (PMC9601815; doi:10.3390/genes13101861)
Supplement: Supplementary file 1 [file genes-13-01861-s001.zip › genes-1943280-supplementary.pdf]

genes

MDPI

Table S1

PCR primers used for RT-PCR and qRT-PCR analyses.

For cloning 3UTR of *orb2*	
Orb2-F	CCCTCGAGAAATAGCAATGCGTCAGGTG
Orb2-R	TTGCGGCCGCAGCTGCTGTACACTATTCTCA
For dsRNA synthesis	
Orb2-F	GGATCCTAATACGACTCACTATAGGCCTACTTGGATGGCAATATGA
Orb2-R	GGATCCTAATACGACTCACTATAGGATAGCAAACACCACCATACA
dsGFP-F	GGATCCTAATACGACTCACTATAGGATACGGCGTGCAGTGCT
dsGFP-R	GGATCCTAATACGACTCACTATAGGATGATCGCGCTTCTCG
For-qRT PCR	
orb2-F	ATGTAAGCGCCTATCATGTG
orb2-R	AGCTTGCGATCCGTTATATG
miR-125-3p	ACAAGTTTTGATCTCCGGTAT
miR-276b-3p	TAGGAACTTAATACCGTGCTCT
Actin-F	CGTTTCCGTTGCCCAGAATTCC
Actin-R	TCAGCAATACCTGGGTACATG
U6	AGGATGACACGCAAAATCGT

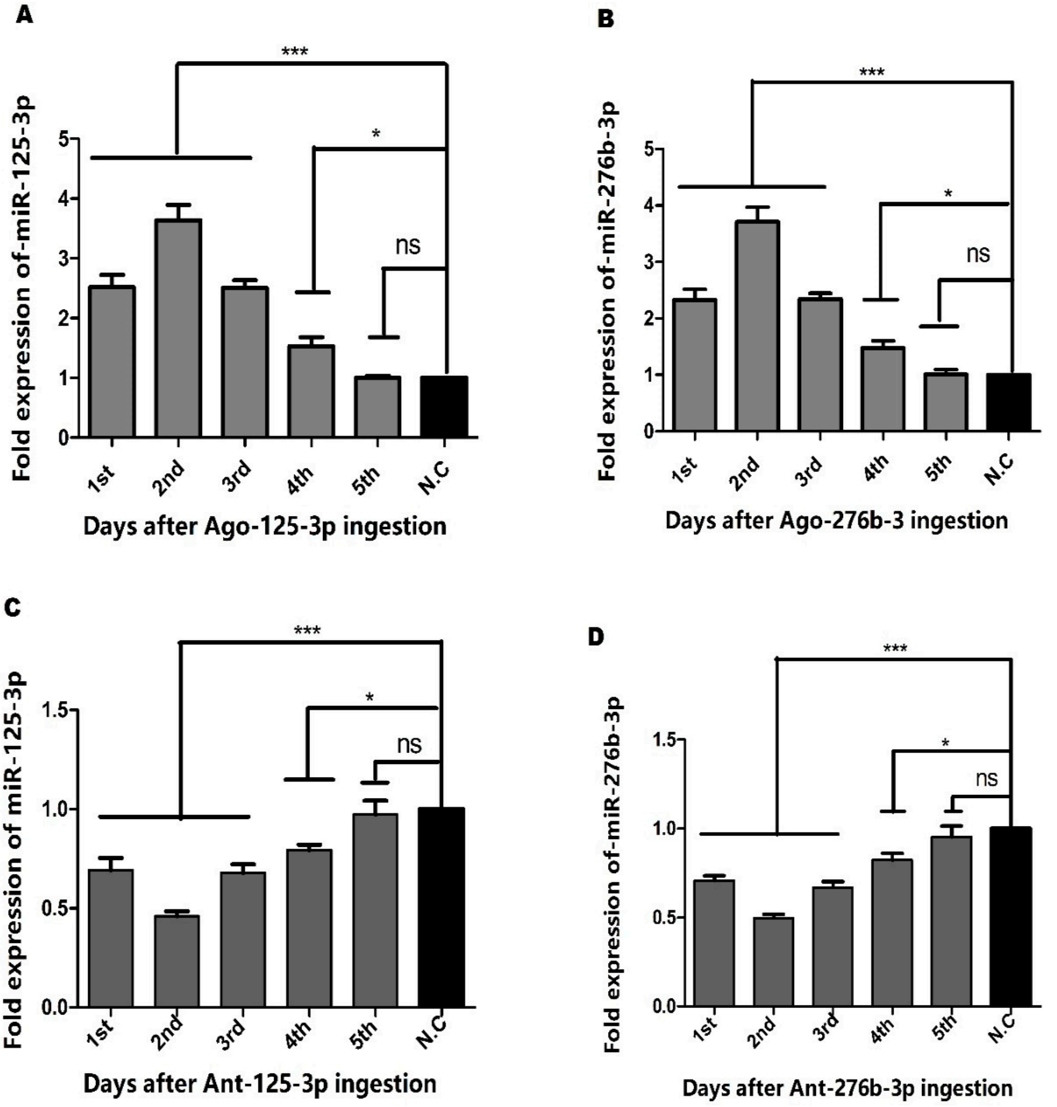

Figure S1: Fold expression of miR-125-3p and miR-276b-3p after feeding of agomirs and antagomirs(A) Fold expression of miR-125-3p after ingestion of agomir-125-3p. (B) Fold expression of miR-276b-3p after ingestion of a mir-276b-3p. (C) Expression miR-125-3p after ingestion of antagomir-125-3p.(D) Fold expression of miR-276b-3p after ingestion of antagomir-276b-3p (N. C) Represents Negative Control Three independent biological replicates were performed. One way Anova was used to analyze the results ($P < 0.0001$, Tuckey test). miR-125-3p and miR-276b-3p expression was nor malized to U6.
